# Health system’s readiness to provide cardiovascular, diabetes and chronic respiratory disease related services in Nepal: analysis using 2015 health facility survey

**DOI:** 10.1186/s12889-020-09279-z

**Published:** 2020-07-25

**Authors:** Umesh Ghimire, Nipun Shrestha, Bipin Adhikari, Suresh Mehata, Yashashwi Pokharel, Shiva Raj Mishra

**Affiliations:** 1 Kalopul, Rudramati Marga, Kathmandu, 44600 Bagmati Nepal; 2 Institute for Health and Sport (IHeS), Victoria University, Melbourne, Australia; 3 Nepal Community Health and Development Centre, Kathmandu, Nepal; 4 Centre for Tropical Medicine and Global Health, Nuffield Department of Medicine, University of Oxford, Oxford, OX1 3SY UK; 5 Ministry of Health and Population, Kathmandu, Nepal; 6 Health Foundation Nepal, America Nepal Medical Foundation, Kathmandu, Nepal; 7 University of Missouri, Kansas City, USA; 8 Nepal Development Society, Bharatpur, Chitwan Nepal

**Keywords:** Service availability, Services readiness, Health system, Non-communicable diseases, Cardiovascular diseases, Diabetes, Chronic respiratory disease

## Abstract

**Background:**

The burgeoning rise of non-communicable diseases (NCDs) is posing serious challenges in resource constrained health facilities of Nepal. The main objective of this study was to assess the readiness of health facilities for cardiovascular diseases (CVDs), diabetes and chronic respiratory diseases (CRDs) services in Nepal.

**Methods:**

This study utilized data from the Nepal Health Facility Survey 2015. General readiness of 940 health facilities along with disease specific readiness for CVDs, diabetes, and CRDs were assessed using the Service Availability and Readiness Assessment manual of the World Health Organization. Health facilities were categorized into public and private facilities.

**Results:**

Out of a total of 940 health facilities assessed, private facilities showed higher availability of items of general service readiness except for standard precautions for infection prevention, compared to public facilities. The multivariable adjusted regression coefficients for CVDs (β = 2.87, 95%CI: 2.42–3.39), diabetes (β =3.02, 95%CI: 2.03–4.49), and CRDs (β = 15.95, 95%CI: 4.61–55.13) at private facilities were higher than the public facilities. Health facilities located in the hills had a higher readiness index for CVDs (β = 1.99, 95%CI: 1.02–1.39). Service readiness for CVDs (β = 1.13, 95%CI: 1.04–1.23) and diabetes (β = 1.78, 95%CI: 1.23–2.59) were higher in the urban municipalities than in rural municipalities. Finally, disease-related services readiness index was sub-optimal with some degree of variation at the province level in Nepal. Compared to province 1, province 2 (β = 0.83, 95%CI: 0.73–0.95) had lower, and province 4 (β =1.24, 95%CI: 1.07–1.43) and province 5 (β =1.17, 95%CI: 1.02–1.34) had higher readiness index for CVDs.

**Conclusion:**

This study found sub-optimal readiness of services related to three NCDs at the public facilities in Nepal. Compared to public facilities, private facilities showed higher readiness scores for CVDs, diabetes, and CRDs. There is an urgent need for policy reform to improve the health services for NCDs, particularly in public facilities.

## Background

Non-communicable diseases (NCDs) are the leading causes of disability adjusted life years (DALYs) and mortality in recent years in Nepal [1]. According to the Global Burden of Diseases, nearly 82,976 deaths in Nepal in 2017 were reported due to NCDs [2]. NCDs collectively contributed to 75 to 82% of total DALYs in 2017 [1]. Almost 80% of the patients attending outpatient departments in Nepal have at least one NCDs and around half of the deaths are due to NCDs [3]. Available data suggest nearly one-third of Nepalese develop hypertension and one-sixth develop diabetes, and this is thought to be underestimated [3].

Substandard health care and low coverage, mostly due to urban centric health services for the management of NCDs can contribute to higher overall disease and disability burden. To combat the rising burden of NCDs, the government of Nepal has developed a multi-sectoral plan for the prevention and control of NCDs in 2014 [4]. Although funds have been allocated for prevention and management of NCDs in Nepal, program implementation has not been initiated even after 5 years of its inception. Health services in relation to the diagnosis and treatment of NCDs is a demanding undertaking that requires efficient health care system, investment, and surveillance [5]. Nepal’s health system suffers from several constraints such as poor and unequal health care services, poor infrastructure, inefficient supply-chain logistics (inadequate supply of essential medicines and equipment) with inadequate human resources and their poor retention [6, 7]. A comprehensive assessment of private and public health facilities is thus essential to identify the capacity of health facilities to deliver quality NCDs screening and treatment services.

Nepal’s health system is heavily dependent on out of pocket (OOP) health spending. Nearly 70% of health expenditure in Nepal is contributed by OOP [8]. Private sector contributes the bulk of specialized health services, and its contribution to the total share of health services is growing. From 1995 to 2008, the number of private hospitals grew by 78% compared to a mere 23% increase in public sector [9]. Approximately, 70% of the total health expenditure in Nepal was estimated from private health facilities, of which 85% were out of pocket [10]. However, there are very few studies exploring the readiness of private sectors to provide quality health services. Understanding of systematic differences in service provisions between private and public health service providers in Nepal will be essential to inform health policy, planning and implementation.

**Fig. 1:**
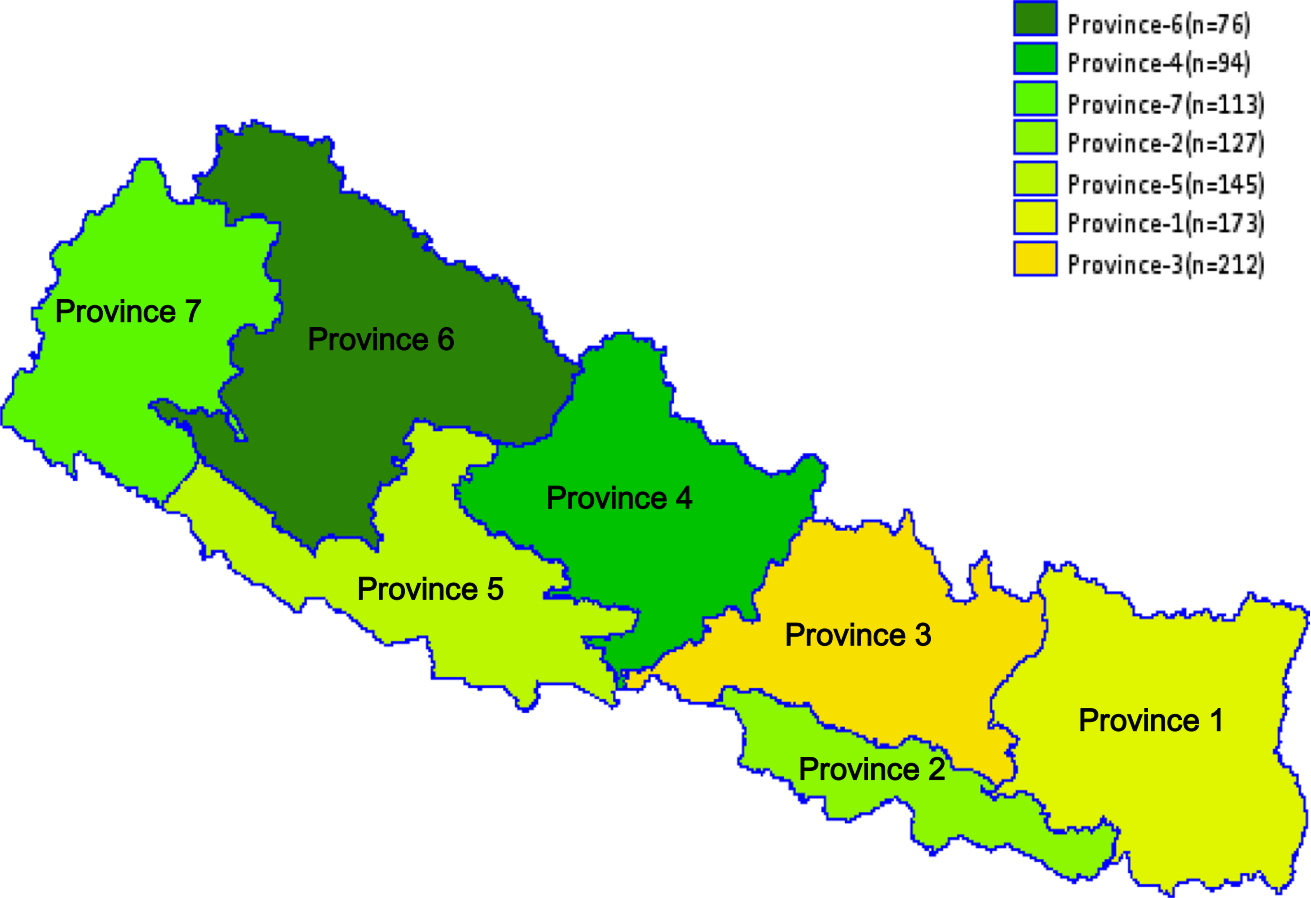
Number of health facilities included in the current study by provinces. Geospatial map was created using GMAP procedure in SAS 9.4. The shape files were obtained from the Government of Nepal, Ministry of Federal Affairs and Local Development and were publicly available for unrestricted use (https://data.humdata.org/dataset/admin-shapefiles-of-nepal-mofald)

**Fig. 2:**
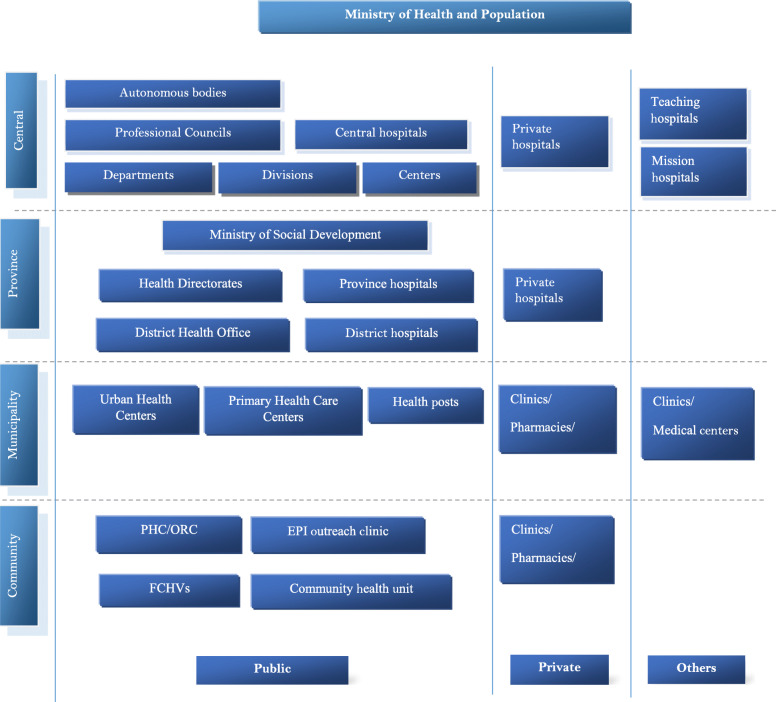
Organization of Nepal’s health system. The figure was developed by one of the authors (SRM) based on the information available from Nepal’s Ministry of Health and Population (https://mohp.gov.np/eng/)

With the promulgation of new constitution in 2015 and transformation of Nepal into federal republic from unitary system, the country has been restructured into seven provinces. The seven provinces are sub-divided into 753 local governments comprising six metropolitan cities, 11 sub-metropolis, 276 urban municipal councils and 460 rural municipalities [11]. The health system in Nepal has also been restructured in alignment with three tiers of government (Figs. 1 and 2). These changes have paved a path for new opportunities for better health systems and have also uncovered many new challenges such as severe depletion of health professionals [12].

The WHO has set a target strategy to reduce 25% of premature mortality due to NCDs by 2025 [13], however, health systems in developing countries such as Nepal face myriad challenges in providing services to prevent and treat NCDs [14–16]. There have not been any studies in the past exploring the challenges and readiness of health systems in tackling the NCDs in Nepal. Readiness of the health system for NCDs is defined as the ability of health system to provide services to these diseases [17]. Readiness assessment is important for benchmarking the coverage and quality of health system and supporting policy-makers in planning appropriate sustainable responses [17]. The main objective of this study was to assess the readiness of health facilities for CVDs, diabetes, and CRDs services in a representative sample of public and private health facilities across all seven provinces in Nepal.

## Methods

### Nepal’s health system

Nepal’s health system operates under the stewardship of Ministry of Health and Population (MoHP). Nepal’s health structures can be divided into federal, provincial, and local levels. The public and federal hospitals are at the top tier to provide tertiary health care. Provincial and district hospitals are the main centers for tertiary care in a province. At the bottom tier are community level health centres such as primary health care centres (PHCCs), urban health centers, and health posts (HPs). At the community level, outreach centers for expanded programme on immunization (EPI), maternal and neonatal child health (MNCH) programme, and family planning (FP) programme are functional as mobile clinics. Moreover, female community health volunteers (FCHVs) are available at the local level both in urban and rural settings in order to facilitate health promotion activities such as conducting mothers’ group meetings; and distributing drugs such as Vitamin A, Diethylcarbamazine for lymphatic filariasis, and refilling condoms & contraceptive pills. All tiered public hospitals have parallel private sector health care institutions.

### Study design

This study used data from the 2015 Nepal Health Facility Survey (NHFS). The 2015 NHFS was the first nationally representative, cross-sectional survey of health facilities across all seven provinces in Nepal. The survey involved a total of 963 health facilities, selected randomly from a list of 4719 health facilities from 13 sample domains, considering equal proportions of health facilities from each stratum. The final sample of health facilities comprised of all non-specialized government hospitals, all private hospitals with at least 100 inpatients beds, and all PHCCs. The remaining health facilities were health posts, private hospitals with at least 15 beds, stand-alone HIV testing and counseling (HTC) sites, and urban health centers (UHCs). The 2015 NHFS collected information on the availability of health services including availability of basic services, human resources, logistics, essential drugs, laboratory services, and infection control mechanisms following standard procedures in health facilities. The data of 2015 NHFS is publicly available in the web portal of DHS program [18], and can be obtained freely after registering for a particular study. The detailed information on the main objectives and survey methodology of 2015 NHFS is published elsewhere [19].

### Data collection tools

The data in the NHFS were collected using comprehensive tools of the Service Availability and Readiness Assessment (SARA) endorsed by the World Health Organization (WHO), Nepal-Service Tracking Survey (STS) by the UK Aid, Facility Assessment for Reproductive Health Commodities Security (FARHCS) by the United Nations Population Fund (UNFPA), and the United States Agency for International Development (USAID)-supported Service Provision Assessment (SPA) survey of the Demographic and Health Survey (DHS) Programme [19]. More information about these tools are available elsewhere [19]. The facility inventory tool was used to collect information on general and specific service availability at the health facilities. The information related to the qualifications, training, clinical experience, level of education, supervision received and perceptions of the service delivery environment from a sample of healthcare providers were recorded in the provider dataset. The survey tools were adapted, validated, and pretested in the Nepalese context [19].

### Data collection

Data collection of 2015 NHFS was performed in a computer-assisted personal interviewing (CAPI) programs after three weeks of training and two days of pre-testing. A total of 86 interviewers with experience as health assistants, executed the data collection of this survey. Eight trainers were assigned to supervise twenty field teams and to monitor data quality. The first phase of data collection took place in April 20 through 25, 2015 and resumed on June 4 after the earthquakes and continued through November 5, 2015. Once data were collected in a facility, they were entered in a tablet computer. The collected data were then transferred to a secured server after a team leader conducted consistency and structural checks to identify any errors or missing information.

### Data analysis

This study used two datasets from the 2015 NHFS: data from the facility inventory dataset and provider dataset. The study excluded 23 HTCs since these facilities were not supposed to provide services related to CVDs, diabetes, and CRDs. A total of 940 health facilities reporting complete data were included in the final analysis. General service readiness of health facilities was assessed in five domains (e.g. basic amenities, basic equipment, standard precautions for prevention of infection, diagnostic capacity and essential medicine). The analyses specific to service readiness to CVDs, diabetes, and CRDs were carried out following the WHO’s SARA manual [17].

Readiness indicators for each item under main service domain were recoded as binary variables, taking value “1” for the availability of tracer item and “0” for the absence of items in the facility. Findings on general service readiness and disease-specific readiness (Supplementary Table 1) were disaggregated into public facilities and private facilities. Altogether, 940 health facilities were included to calculate the service readiness of diabetes-related, CVD-related, and CRD-related readiness score in this study. Availability of basic health services, human resources, logistics, laboratory services and essential medicine were assessed using the WHO-SARA scoring guideline for health services readiness for NCDs [20]. Service readiness scores range between 11.3 to 34.7; higher score reflects better preparedness. Detailed description of each domain is presented in Supplementary Table 1. Level of urbanization was assigned to each health facilities based on government classification: urban and rural categories. We aggregated metropolitan city and sub-metropolitan city into one, considering it as the highest level of urbanization followed by urban municipality and rural municipality.

The primary objective of this study was to assess readiness of public and private health facilities that provide services for CVDs, diabetes, and CRDs in Nepal. This was initially accomplished using raw readiness scores followed by multivariable models. Following the WHO-SARA guideline of health facilities [17], mean and standard deviation (±SD) of all domain raw scores were calculated for general, CVDs, diabetes, and CRDs-related service readiness index. CRD-related service readiness index is based on the mean availability of items as percentage within that domain. The distributions of readiness indices specific to CVDs, diabetes, and CRDs were negatively skewed. Hence, we calculated median (Q1, Q3) to express the readiness of health facilities.

The secondary objective of this study was to understand whether heterogeneity in readiness exist in different regions of Nepal. This was assessed using median readiness index for these diseases stratified by seven provinces, facility type (public and private) and the level of urbanization (metropolitan, urban, and rural municipality). In addition, multiple linear regression analysis was used to account for potential confounders of service readiness by adjusting for health facility type, ecological region and level of urbanization. The dependent variables were log-transformed before the analysis and regression coefficient (β) was calculated based on the antilog of unstandardized regression coefficient obtained from the analysis. Beta coefficient ≥ 1 from regression models denotes favourable changes in readiness index and vice-versa. The outcome variables were log-transformed before analysis to address the non-normal distribution of residuals detected in the regression models. A two-sided *p*-value below 0.05 was considered as statistically significant. All analyses were conducted using STATA version 15 (StataCorp LP, College Station, TX, USA) [21] and were adjusted for sample weight.

## Results

Findings of general service readiness and mean domain score of items are shown in Table 1. Out of a total of 940 health facilities, majority (870; 93%) were public health facilities. The mean domain scores for basic amenities, basic equipment, standard precautions, laboratory capacity, and essential medicines were 53.7 (±SD 21.3), 77.2 (±SD 17.6), 59.2 (±SD 19.1), 16.6 (±SD 30.0), and 33.3 (±SD 15.5), respectively. Except for standard precautions for infection prevention, private facilities possessed a higher availability of items in four domains of general service readiness. Amongst all domains, mean domain score in public facilities was very low for diagnostic capacity. Overall, median readiness index comprising all domains was 53.8 (Q1, Q3: 43.7, 69.4). Private facilities had a higher median readiness index 75.2 (65.8, 84.3) compared to public facilities 50.3 (Q1, Q3: 42.4, 63.3).

**Table 1:** Status of general service readiness indicators of the health facilities

General readiness	Public facilities ***n*** = 870	Private facilities ***n*** = 70	Total ***n*** = 940
**Basic amenities**			
Power	44.3	99.4	48.4
Water source	80.0	89.4	80.7
Room with privacy	76.7	95.7	78.1
Adequate sanitation facilities	80.0	98.4	81.4
Communication equipment	12.1	98.4	18.5
Access to computer with internet	4.2	78.7	9.7
Emergency transportation (ambulance)	56.4	94.5	59.2
**Mean domain score of basic amenities (±SD)**	50.5 (18.6)	93.5 (9.6)	53.7 (21.3)
**Basic equipment**			
Blood pressure apparatus	94.0	95.6	94.1
Stethoscope	97.9	96.7	97.8
Adult scale	88.2	94.1	88.6
Child scale	40.3	25.6	39.2
Thermometer	92.8	96.4	93.0
Light source	47.6	88.8	50.7
**Mean domain score of basic equipment (±SD)**	76.8 (17.5)	82.9 (18.3)	77.2 (17.6)
**Standard precautions for infection prevention**			
Safe final disposal of sharps	84.0	85.4	84.2
Safe final disposal of infectious wastes	81.5	74.6	81.0
Appropriate storage of sharps waste	81.1	34.0	77.6
Appropriate storage of infectious waste	4.8	4.7	4.8
Disinfectant	62.4	63.1	62.5
Single-use, standard disposable or auto-disable syringes	84.2	60.1	82.4
Soap and running water or alcohol-based hand rub	55.8	73.9	57.1
Disposable latex gloves	79.6	83.0	79.9
Guidelines on standard precautions	3.3	5.3	3.5
**Mean domain score of standard precautions for infection prevention (±SD)**	59.6 (18.8)	53.8 (21.9)	59.2 (19.1)
**Diagnostic capacity**			
Blood glucose test	3.7	58.0	7.7
Hemoglobin test	9.3	93.3	15.5
HIV diagnostic capacity	8.9	87.1	14.7
Malaria diagnostic capacity	18.6	95.3	24.3
Syphilis RDT	5.6	78.1	11.0
Urine test for pregnancy	25.4	90.0	30.2
Urine dipstick- protein	9.8	90.5	15.8
Urine dipstick- glucose	7.7	91.8	14.0
**Mean domain score of diagnostic capacity (±SD)**	11.1 (22.9)	85.5 (21.5)	16.6 (30.0)
**Essential medicines**			
Amitriptyline tablet	4.7	58.3	9.8
Amlodipine tablet or alternative calcium channel blocker	5.1	68.3	11.2
Amoxicillin syrup/suspension or dispersible tablet	21.6	57.4	24.2
Amoxicillin tablet	89.6	71.4	88.3
Ampicillin powder for injection	4.0	36.7	6.4
Beclometasone inhaler	3.2	33.0	6.0
Ceftriaxone injection	18.0	43.6	19.9
Enalapril tablet or alternative ACE inhibitor e.g. lisinopril, ramipril, perindopril	13.9	57.0	18.0
Fluoxetine tablet	NA	NA	NA
Gentamicin injection	63.5	64.4	63.6
Glibenclamide tablet	1.4	30.3	4.2
Ibuprofen tablet	18.0	74.3	22.2
Insulin regular injection	4.3	50.8	19.9
Metformin tablet	2.6	67.8	7.5
Omeprazole tablet or alternative such as pantoprazole, rabeprazole	46.2	72.8	48.2
Oral Rehydration Solution (ORS)	93.3	77.8	92.2
Paracetamol tab/injection	99.4	73.3	97.4
Salbutamol tab or inhaler	78.2	68.0	77.4
Simvastatin tablet or other statin e.g. atorvastatin, pravastatin, fluvastatin	0.6	19.5	2.4
Zinc sulphate tab	98.1	61.3	95.4
**Mean domain score of items of essential medicine (±SD)**	31.4 (10.6)	56.0 (35.7)	33.3 (15.5)
**General services readiness (Median (Q1, Q3))**	50.3 (42.4, 63.3)	75.2 (65.8, 84.3)	53.8 (43.7, 69.4)

Fluoxetine tablet was not available in the dataset

Ibuprofen tablet was not available in the NHFS dataset, Diclofenac was used instead

Enalapril tablet or alternative ACE inhibitor e.g. lisinopril, ramipril, perindopril was not available in the NHFS dataset, Atenolol was used instead

### Readiness index specific to services for CVDs

In total, 940 health facilities provided information on availability of diagnosis and treatment facility (Table 2). The mean domain score on the availability of guidelines on diagnosis and treatment and trained staff on CVDs treatment was very low; 1.4 and 1.3 respectively. The mean domain index of equipment in the health facilities that provide CVD services was 68.2 (±SD 21.1). The mean domain index for CVD medicines was as low as 5.4 (±SD 15.5) and availability of essential medicines for CVD was low in public facilities. The overall median readiness index for CVDs was 18.8 (Q1, Q3: 18.8, 25.0).

**Table 2:** Readiness index scores specific to services for CVD and domain scores by facility type

Services for CVDs	Public facilities ***n*** = 870	Private facilities ***n*** = 70	Total ***n*** = 940
Diagnosis and treatment facilities	71.3	94.6	73.1
Guidelines on diagnosis and treatment^a^	1.4	1.3	1.4
**Mean guidelines domain index (±SD)**	1.4	1.3	1.4
Trained staff^a^	1.4	3.1	1.3
**Mean trained staff domain index (±SD)**	1.4	3.1	1.3
**Equipment**^a^			
Stethoscope	97.9	96.9	97.8
Blood pressure	93.5	95.7	93.7
Adult weighing scale	87.0	93.5	87.6
Oxygen	4.4	55.1	9.3
**Mean equipment domain index (±SD)**	67.1 (20.2)	81.2 (27.4)	68.2 (21.1)
**Medicines**^a^			
Amlodipine/nifedipine	5.1	68.3	11.2
Beta-blockers (atenolol)	13.9	57.0	18.0
Aspirin	4.2	63.5	9.9
Thiazide	2.1	26.2	4.4
**Mean medicines domain index (±SD)**	2.6 (9.8)	39.1 (29.0)	5.4 (15.5)
**Readiness index specific to services for CVD (Median (Q1, Q3))**	18.8 (18.8, 18.8)	31.3 (18.9, 37.5)	18.8 (18.8, 25.0)

^a^This analysis is limited to a sub-sample of Public facilities (*n* = 621), Private facilities (*n* = 66) and all combined (*n* = 687)

### Readiness index specific to services for diabetes

Among all health facilities, the majority of private facilities offered diagnosis and treatment of diabetes (Table 3). The mean domain score for the availability of guidelines on the diagnosis and treatment for diabetes was 4.1 and less than 2 for the trained health personnel available at the facilities. The mean domain index of availability of equipment that offered diabetes services was 40.4 (±SD 24.4). The mean domain index of diagnostic capacity and medicine was 9.0 (±SD 24.3) and 11.1 (±SD 20.7), respectively, and the median readiness index for diabetes was 26.4 (Q1, Q3: 20.8, 33.3).

**Table 3:** Readiness index scores specific to services for diabetes and domain scores by facility type

Services for diabetes	Public facilities ***n*** = 870	Private facilities ***n*** = 70	Total ***n*** = 940
Diagnosis and treatment facilities	15.1	95.2	21.1
Guidelines for diagnosis and treatment^a^	4.8	2.5	4.1
**Mean guidelines domain index**	4.8	2.5	4.1
Trained staff^a^	1.5	2.5	1.9
**Mean staff domain index**	1.5	2.5	1.9
**Equipment**^a^			
Blood pressure	93.5	95.7	94.2
Adult weighing scale	84.8	93.5	87.7
Height board/stadiometer	26.7	35.9	29.8
**Mean equipment domain index (±SD)**	37.9 (22.3)	71.4 (27.5)	40.4 (24.4)
**Diagnostic capacity**^a^			
Blood glucose	5.5	24.7	11.9
Urine protein	39.7	82.2	53.9
Urine glucose	40.2	85.1	55.3
**Mean diagnostics domain index (±SD)**	4.7 (17.6)	63.1 (30.5)	9.0 (24.3)
**Medicines**^a^			
Metformin	16.5	69.5	34.3
Glibenclamide	6.4	30.1	14.3
Injectable insulin	4.3	50.8	19.9
Injectable glucose solution	46.7	69.9	54.5
**Mean medicines domain index (±SD)**	7.7 (13.7)	54.4 (38.0)	11.1 (20.7)
**Readiness index specific to services for diabetes (Median (Q1, Q3))**	26.4 (16.7, 30.6)	34.7 (26.4, 40.3)	26.4 (20.8, 33.3)

^a^This analysis is limited to a sub-sample of Public facilities (*n* = 132), Private facilities (*n* = 66) and all combined (*n* = 198)

### Readiness index specific to services for chronic respiratory diseases

Out of 940 health facilities, nearly 5% did not provide service for diagnosis and treatment for CRDs. The mean domain scores of availability of guidelines and trained provider related to CRDs were 4.6 and 9.0, respectively. Availability of both equipment and medicines was higher in private facilities. The overall median readiness index specific to services for CRDs service was 11.3 (Q1, Q3: 11.3, 18.8) (Table 4).

**Table 4:** Readiness index scores specific to services for chronic respiratory diseases and domain scores by facility type

Services for chronic respiratory diseases	Public facilities *n* = 870	Private facilities *n* = 70	Total *n* = 940
Both diagnosis and treatment facilities	94.1	94.9	94.1
Guidelines on the diagnosis and treatment^a^	4.9	0.6	4.6
**Mean guidelines domain index**	4.9	0.6	4.6
Trained staff^a^	9.4	3.4	9.0
**Mean staff domain index**	9.4	3.4	9.0
**Equipment**^**a**^			
Stethoscope	97.8	96.9	97.7
Oxygen flow meter	2.5	49.3	6.0
Spacers for inhalers	2.1	25.0	3.8
Oxygen	3.4	54.9	7.2
**Mean equipment domain index (±SD)**	25.0 (11.4)	53.9 (33.7)	27.2 (16.1)
**Medicines**^**a**^			
Salbutamol inhaler	79.7	68.2	78.8
Beclomethasone inhaler	3.0	32.9	5.3
Prednisolone cap/tabs	3.4	64.9	8.0
Hydrocortisone injection	6.9	69.9	11.6
Epinephrine injectable	5.2	59.1	9.2
**Mean medicines domain index (±SD)**	19.3 (14.3)	57.9 (39.6)	22.2 (20.2)
**Readiness index specific to services for chronic resp. (Median (Q1, Q3))**	11.3 (11.3, 16.3)	26.3 (6.5, 37.5)	11.3 (11.3, 18.8)

^a^This analysis is limited to a sub-sample of Public facilities (*n* = 819), Private facilities (*n* = 66) and all combined (*n* = 885)

Table 5 shows the multiple linear regression for CVDs, diabetes and CRDs-specific service readiness index by province, facility type, ecological region, and level of urbanization. There were no major differences in provinces except for lower CVDs-specific readiness noted in province 2 (β =0.83, 95%CI: 0.73–0.95), and higher CVDs-specific readiness noted in province 4 (β =1.24, 95%CI: 1.07–1.43) and province 5 (β =1.17, 95%CI: 1.02–1.34) compared to province 1. When assessed by health facility type, private facilities had significantly higher readiness compared to public facilities for CVDs (β =2.87, 95%CI: 2.42–3.39, diabetes (β =3.02, 95%CI: 2.03–4.49), and CRDs (β =15.95, 95%CI: 4.61–55.13). Service readiness index by ecological region showed that health facilities in Hills were far better than in Terai for CVDs (β =1.99, 95%CI: 0.91–1.11). Urban municipalities had a higher service readiness score than rural municipalities for CVDs (β =1.13, 95%CI: 1.04–1.23) and diabetes (β =1.78, 95%CI: 1.23–2.59).

**Table 5:** Multiple regression analyses of health facility characteristics with the service readiness index

	CVDs^**a**^		Diabetes^**a**^		CRDs^**a**^	
	Adjusted β (95%CI)	*p*-value	Adjusted β (95%CI)	*p*-value	Adjusted β (95%CI)	*p*-value
**Province**						
Province 1	**ref**		**ref**		**ref**	
Province 2	0.83 (0.73–0.95)	0.01	0.94 (0.53–1.66)	0.89	1.18 (0.87–1.59)	0.18
Province 3	1.05 (0.92–1.19)	0.46	1.38 (0.86–2.23)	0.33	1.29 (0.78–2.12)	0.28
Province 4	1.24 (1.07–1.43)	0.00	1.98 (1.08–3.61)	0.88	1.36 (0.77–2.42)	0.32
Province 5	1.17 (1.02–1.34)	0.02	1.21 (0.73–2.01)	0.22	1.36 (0.77–2.42)	0.29
Province 6	0.01 (0.85–1.21)	0.87	1.15 (0.58–2.29)	0.83	0.73 (0.36–1.48)	0.38
Province 7	0.06 (0.91–1.24)	0.47	1.34 (0.73–2.45)	0.33	1.25 (0.50–3.13)	0.63
**Health facility type**						
Public facilities	**ref**		**ref**		**ref**	
Private facilities	2.87 (2.42–3.39)	0.00	3.02 (2.03–4.49)	0.00	15.95 (4.61–55.13)	0.00
**Ecological region**						
Mountains	1.88 (0.78–0.99)	0.68	1.19 (−0.14–0.9)	0.49	1.07 (0.89–1.30)	0.47
Hills	1.99 (0.91–1.11)	0.03	1.15 (0.78–1.707)	0.46	1.13 (0.97–1.32)	0.11
Terai	**ref**		**ref**		**ref**	
**Urbanization**						
Metropolitan	0.99 (0.80–1.22)	0.92	1.59 (0.89–2.85)	0.12	0.80 (0.59–1.09)	0.16
Urban Municipality	1.13 (1.04–1.23)	0.01	1.78 (1.23–2.59)	0.00	1.02 (0.90–1.16)	0.76
Rural Municipality	**ref**		**ref**		**ref**	

^a^dependent variable were log-transformed before analysis

^β^regression coefficient was calculated based on the antilog of unstandardized regression coefficient. β of ≥1 denotes favourable changes in readiness index while assessing the relationship with the predictor variable. The model is adjusted for health facility types, ecological region and urbanization

^CI^confidence interval (unstandardized regression coefficient)

### Readiness index according to provinces, health facility types, ecological region and levels of urbanization

**Fig. 3:**
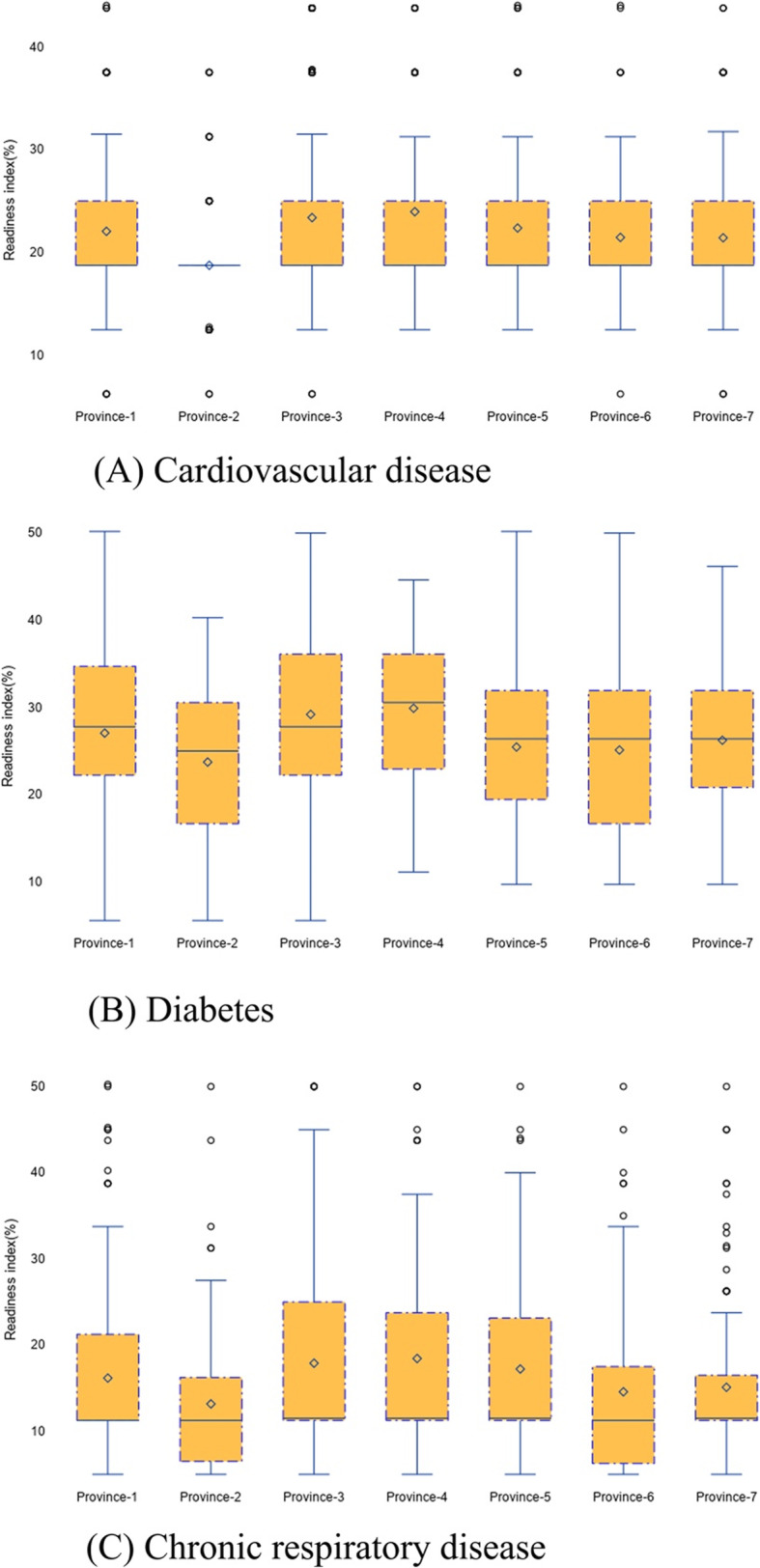
Differences between the service readiness indexes in health facilities across seven provinces (The figure shows the medians and interquartile range for cardiovascular, diabetes, and chronic respiratory disease-specific readiness index at the province level in Nepal)

The median readiness index for CVDs, diabetes, and CRDs in public health facilities in Nepal was very low ranging between 9.2 to 27.9 (Fig. 3). The service readiness score was particularly low in public facilities for CVDs and CRDs.

**Fig. 4:**
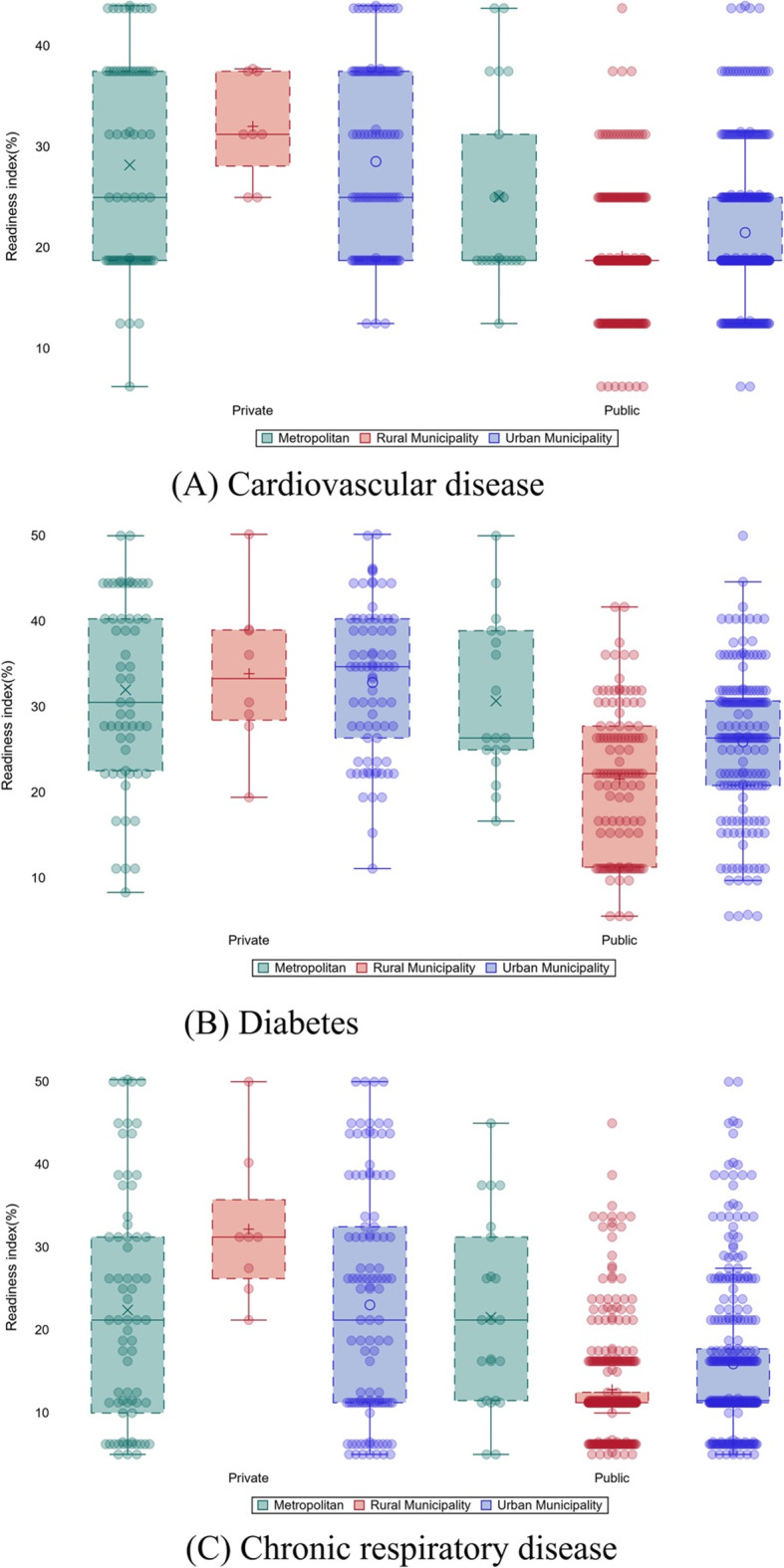
Differences between the service readiness indexes of metropolitan, municipality, and rural health facilities

Overall, the median readiness index for CVDs, diabetes, and CRDs were low (less than 30%), with little heterogeneity across the seven provinces. Compared to the median values for diabetes, the median values for CVDs, and CRDs were consistently low in Province 2, Province 6 and Province 7 (Fig. 4).

The median readiness index by urbanization (metropolitan, urban and rural municipality) and health facility type is shown in Fig. 4. In general, the overall median readiness index was less than 40 for CVDs, diabetes, and CRDs across these categories. However, the readiness was even lower for public health facilities irrespective of urbanization or disease.

## Discussion

### Overall findings

Current study extracted from a nationally representative sample of 940 health facilities shows that majority of health facilities across all seven provinces had sub-optimal readiness to manage CVDs, diabetes, and CRDs based on WHO SARA guideline [13]. Against the backdrop of the WHO’s target strategy to reduce 25% of premature mortality or preventable deaths due to NCDs by 2025 [13], health systems in developing countries such as Nepal face significant challenges in providing services for the prevention and treatment of NCDs [14–16]. Private health facilities were better equipped to provide services related to CVDs, than public health facilities. Most of the facilities lacked trained human resources, equipment, drugs, and standard guidelines for effective NCDs care and management.

### Readiness of public and private health facilities for NCDs

Compared to the service readiness of private health facilities, the readiness scores for public health facilities was low. There is an increasing trend to visit private health facilities in Nepal for the pursuit of better health care, particularly patients from average to high socio-economic status [10, 22]. Healthcare expenditure in Nepal is mostly out of pocket and constitutes one-third of the total expenses involving both private and public hospitals [23]. In such a context, lack of readiness of public facilities where patients resort for quality health care, poses a major challenge in diagnosis of NCDs and its management. Similar findings have been found in other resource constrained settings of low- and middle- income countries (LMICs) [24, 25]. In general, driven by the lack of political stability and economic constraints, LMICs face significant challenges in maintaining preparedness of health system, coverage, and quality of care.

One of the major challenges in Nepal’s health system is the disproportionate lack of human resources, medicines, equipment, and supply chain logistics in remote regions of Nepal [6, 7, 26]. In addition, other factors such as patient’s socio-economic status, distance to health centres, transportation, direct, and indirect costs associated with attending health centres further compound the utilization of health services in rural regions of Nepal [7, 27], and resonate with other LMICs [28]. Standing at the forefront of health services, health care providers, particularly in PHCCs and HPs, significantly lack adequate training and experiences in the management of NCDs echoing with the health systems in sub-Saharan Africa [29]. Even if human resources were ready to serve the patients, the health facilities often lack simple diagnostic materials, equipment such as glucometer or a basic lab equipment to measure blood glucose level.

Most of the public health facilities faced shortfall in the availability of medicines for CVDs and diabetes. Although, the basic diagnostic items such as sphygmomanometer and stethoscope were readily available, unavailability of glucose strips and essential medicines such as blood pressure lowering drugs and anti-diabetics hinder the quality NCDs care by health care providers. Our findings resonate with the studies from LMICs settings of Africa and Asia [30–33]. Despite the ample evidence that essential medicines for NCDs reduce the burden of NCDs, public health facilities often lack essential medicines; and health care is often unaffordable in the private sector, particularly for the population from low socio-economic status.

Management of NCDs requires prolonged follow-ups with regular access to medicines and health care; any impediment to access and care can prompt patients to discontinue their treatment and may fall prey to poly-visits to both formal and informal health care providers. The latter can include visiting traditional healers who often sell unknown chemical compounds [34, 35] and others constitute drug peddlers, locally known as ‘*Jhole doctors’* in Terai region of Nepal [36]. Although these informal drug peddlers are illegal, the ease of access and their local availability can mean that patients rely on their poor diagnostic skills and sub-standard, and counterfeit medications which can delay the healthcare seeking behavior, distort the symptoms and develop complications and death [36]. While WHO advocates for the global priorities in increasing an access to essential, quality-assured, safe, effective, and affordable medical products, countries in LMICs struggle to achieve the universal health coverage [37, 38].

In this study, unavailability of guidelines for early detection, management and prompt referral of CVDs, diabetes, and CRDs; poor monitoring and evaluation system for tracking NCDs; and weak referral linkages between primary and higher health care facilities were found to be the major barriers in NCDs prevention and care. Several studies have reported the low service readiness in health facilities in rural parts of the country compared to the health facilities in urban areas [7, 39, 40]. Similar findings were observed in the current study where many health facilities in rural areas were located in hard to reach areas, and often lacked qualified health workers, with high attrition and lack of policy supporting establishment of health care institutions in the rural regions [26, 41]. Such a chronic shortage of health workforce and resources in the rural regions is likely to persist and can be compounded by the transitioning federal health system of Nepal with high level of unwillingness of health care workers to serve in the rural regions [42]. Although government of Nepal reinforced policy to promote the retention of qualified health human resources particularly doctors in the rural regions, such as through promotion, provision of incentives, opportunities in higher education, in addition to compulsory placement of government funded doctors in the rural settings, the attrition remains a major problem [43, 44]. Chronic shortfall of qualified health human resources in the rural settings are attributed to manifold factors including lack of health infrastructure, shortage of equipment, poor academic/clinical stewardship, and urban centric health care system in Nepal [41, 43–45]. The primary health care centers in rural regions of Nepal thus share the disproportionate burden of scarcity in providing health services.

The supply chain logistics providing essential medicines including equipment in such hard to reach areas is compounded by the poor road condition, seasonal flooding, and landslides. For instance, year round availability of essential medicines in Nepal was 16.6% in health facilities from the Mountains, 57.1% in the Hills, and 52.2% in the Terai [46]. A study in Bangladesh reported that the poor supply chain management for essential medicines affected the management of NCDs in the rural settings [47].

### Implications for health policy and planning

Sub-optimal availability of NCDs services in Nepal has major implications for country’s aims for sustainable development goal-3. Nepal seems inadequately prepared to achieve the “Global action plan for the prevention and control of NCDs 2013-2020” [13] which has an ambitious target to reduce premature cardiovascular mortality by 25% by 2025 [4]. Although the Ministry of Health and Population has set steps and promises towards curbing the current under coverage of health services to rural regions, the multi-sectoral plan on management of NCDs faces challenges intertwined in the current health system’s functioning. Nepal should strive towards ensuring the functional capacities of PHCCs (for example, improving supply chain logistics and provision of adequate number of health human resources, training, capacity development, and addressing attrition) together with stringent policy stewardship to improve NCDs care in both PHCCs and private hospitals.

The current restructuring of health system in Nepal in alignment with federal setup can be an excellent opportunity for strengthening health facilities in delivering NCDs services. With an increased influx of responsibility to the provincial and local government in federal context including revenue collection through taxes on tobacco, alcohol and sugary drinks, financial independence thus achieved can be channeled to the management of NCDs.

In order to improve the retention of qualified health human resources in rural regions, augmenting current policies together with infra-structural development is necessary. For instance, physicians may feel motivated when there is an availability of professional supervision, better opportunities for specialized training in addition to current policies of incentives and compulsory placements. In addition, Nepal can adopt the principles inherent in community engagement [48, 49], wherein community and public-private partnership can serve the population in terms of early diagnosis, treatment, and management. The intervention approaches to reduce NCDs in low resource settings as recommended by the WHO includes early detection and diagnosis that could curtail medical costs, improve quality of life, and productivity in LMICs such as Nepal [16]. Recent evidence of training and mobilization of female community health volunteers in the management of hypertension shed some promising steps for Nepal [50]. Such a strategy could be scaled up together with the partnership of community-public and private health service providers through various means including subsidization of health care services to enhance the current coverage for the management of NCDs. The findings of this study can help the concerned stakeholders and policymakers in devising appropriate policies/programmes to strengthen NCD services delivered through public and private health care facilities in Nepal.

### Strengths and limitations

This is the first study exploring the challenges and readiness of health systems in tackling the NCDs in Nepal and utilized the first nationally representative sample of health facilities across all seven provinces in Nepal, thus the findings from this study are generalizable for all regions of Nepal. The other major strengths of this study are that the medicine, diagnostics, and guidelines availability was recorded based on the observations of health facilities by trained survey enumerators. Although several areas were examined in this study such as availability of medicines, diagnostics, and guidelines, most of these were basic assessments and many other equipment and tools required for management of NCDs such as electrocardiogram and other technologies were not considered. Information were missing on one to several items depending on the domains analyzed. Consequently, the sample size was limited to all non-missing items. Our findings are approximate to the original report of Nepal SPA report, 2015 [51], so discrepancies may have occurred due to partitioning of data for analysis. Although reported data were triangulated by observation and through cross-validation (through multiple respondents), information on qualifications, training, clinical experience, and perceptions of the service delivery environment may have incurred desirability and recall bias. Nevertheless, authors’ experience of health services and review of the literature suggest that these public health services, specifically in rural regions suffer from multitude of constraints.

## Conclusions

This study found sub-optimal readiness of health system for services related to CVDs, diabetes, and CRDs particularly at the public health facilities in Nepal. The availability of services was higher in private health facilities compared to the public health facilities. Geographic variation in service readiness index highlights that some provinces are better prepared to provide NCDs services than other provinces. Given Nepal’s commitment under SDG-3, Global Action Plan on NCDs, and commitments under periodic plans and policies, the country needs to strengthen service delivery platforms while improving the overall readiness of health system through increasing the number of qualified health staff, training and provision of equipment and medicines. In addition, future operational and health system research can explore the scalability and practicalities of community-public-private partnership, such as through training of community volunteers, increased engagement with multi-stakeholders and subsidization of basic amenities for detection, treatment and management of NCDs.

## Supplementary information

**Additional file 1: Supplementary table 1.** Description of each domain (general readiness, cardiovascular diseases readiness, diabetes service readiness, and chronic respiratory diseases readiness).

## Data Availability

All data related to this study are publicly available from the DHS program website [51] and can be retrieved after registering for a specific study.

## Abbreviations

CAPI: Computer-Assisted Personal Interviewing
CI: Confident Interval
CVDs: Cardiovascular diseases
CRDs: Chronic Respiratory Diseases
DALY: Disability Adjusted Life in Years
DHS: Demographic and Health Survey
EPI: Expanded Programme on Immunization
FARHCS: Facility Assessment for Reproductive Health Commodities Security
FCHVs: Female Community Health Volunteers
FP: Family Planning
LMICs: Low and Middle Income Countries
MNCH: Maternal and Neonatal Child Health
NCDs: Non-communicable diseases
NHFS: Nepal Health Facility Survey
OOP: Out Of Pocket
PHCCs: Primary Health Care Centers
PHC/ORC: Primary Health Care/Out Reach Clinic
SARA: Service Availability and Readiness Assessment
SD: Standard Deviation
SDG: Sustainable Development Goals
STS: Service Tracking Survey
UK Aid: United Kingdom Aid
UNFPA: United Nations Population Fund
USAID: United States Agency for International Development
WHO: World Health Organization
