# Supplementary material for: Health system’s readiness to provide cardiovascular, diabetes and chronic respiratory disease related services in Nepal: analysis using 2015 health facility survey
Source: BMC Public Health. 2020 Jul 25;20:1163. doi: 10.1186/s12889-020-09279-z (PMC7382840; doi:10.1186/s12889-020-09279-z)
Supplement: Supplementary file 1 — Additional file 1: Supplementary table 1. Description of each domain (general readiness, cardiovascular diseases readiness, diabetes service readiness, and chronic respiratory diseases readiness). [file 12889_2020_9279_MOESM1_ESM.docx]

**Supplementary table 1 Description of each domain (general readiness, cardiovascular disease readiness, diabetes service readiness and chronic respiratory disease (CRD) readiness)**

| **General readiness** | **CVD service readiness** | **Diabetes service readiness** | **CRD service readiness** |
| --- | --- | --- | --- |
| Please tell me if the following amenities are available at this site today and is functioning | Do providers in this facility diagnose and/or manage CVD? | Do providers in this facility diagnose and/or manage diabetes? | Do providers in this facility diagnose and/or manage CRD? |
| A. Power | A. Yes | A. Yes | A. Yes |
| B. Water source | B. No | B. No | B. No |
| C. Room with privacy | Do you have the national guidelines for the diagnosis and management of CVD? | Do you have the national guidelines for the diagnosis and management of diabetes? | Do you have the national guidelines for the diagnosis and management of CRD? |
| D. Adequate sanitation facilities | A. Yes | A. Yes | A. Yes |
| E. Communication equipment | B. No | B. No | B. No |
| F. Access to computer with internet | Had at least one staff member who  had received in-service training in CVD services during the 24 months before the survey? | Had at least one staff member who had received in-service training in diabetes services during the 24 months before the survey? | Had at least one staff member who  had received in-service training in CVD  services during the 24 months before the  survey? |
| G. Emergency transportation (ambulance) | A. Yes | A. Yes | A. Yes |
|  | B. No | B. No | B. No |
| Please tell me if the following equipment are available at this site today and is functioning | Does this facility have below-listed equipment? | Does this facility have below-listed equipment? | Does this facility have below-listed equipment? |
| A. Blood pressure apparatus | A. Stethoscope | A. Blood pressure | A. Stethoscope |
| B. Stethoscope | B. Blood pressure | B. Adult weighing scale | B. Oxygen flow meter |
| C. Adult scale | C. Adult weighing scale | C. Height board/stadiometer | C. Spacers for inhalers |
| D. Infant scale | D. Oxygen | Does this facility do below-listed testing? | D. Oxygen |
| E. Child scale | Are any of the following medicines for the management of CVD available in the  facility/location today? | A. Blood glucose | Are any of the following medicines for the management of CRD available in the  facility/location today? |
| F. Thermometer | A. Amlodipine/nifedipine | B. Urine protein | A. Salbutamol inhaler |
| G. Light source | B. Beta-blockers (atenolol) | C. Urine glucose | B. Beclomethasone inhaler |
| The following standard precautions are  available at this site today? | C. Aspirin | Are any of the following medicines for the management of diabetes available in the facility/location today? | C. Prednisolone cap/tabs |
| A. Safe final disposal of sharps | D. Thiazide | A. Metformin | D. Hydrocortisone injection |
| B. Safe final disposal of infectious wastes |  | B. Glibenclamide | E. Epinephrine injectable |
| C. Appropriate storage of sharps waste |  | C. Injectable insulin |  |
| D. Appropriate storage of infectious waste |  | D. Injectable glucose solution |  |
| E. Disinfectant |  |  |  |
| F. Single-use, standard disposable or auto-disable syringes |  |  |  |
| G. Soap and running water or alcohol-based hand rub |  |  |  |
| H. Disposable latex gloves |  |  |  |
| I. Guidelines on standard precautions |  |  |  |
| The following laboratory capacity are  available at this site? |  |  |  |
| A Blood glucose test |  |  |  |
| B. Hemoglobin test |  |  |  |
| C. HIV diagnostic capacity |  |  |  |
| D. Malaria diagnostic capacity |  |  |  |
| E. Syphilis RDT |  |  |  |
| F. Urine test for pregnancy |  |  |  |
| G. Urine dipstick- protein |  |  |  |
| H. Urine dipstick- glucose |  |  |  |
| **Essential medicines** |  |  |  |
| 1. Amitriptyline tablet |  |  |  |
| 1. Amlodipine tablet or alternative calcium channel blocker |  |  |  |
| 1. Amoxicillin syrup/suspension or dispersible tablet |  |  |  |
| 1. Amoxicillin tablet |  |  |  |
| 1. Ampicillin powder for injection |  |  |  |
| 1. Beclometasone inhaler |  |  |  |
| 1. Ceftriaxone injection |  |  |  |
| 1. Enalapril tablet or alternative ACE inhibitor e.g. lisinopril, ramipril, perindopril |  |  |  |
| 1. Fluoxetine tablet |  |  |  |
| 1. Gentamicin injection |  |  |  |
| 1. Glibenclamide tablet |  |  |  |
| 1. Ibuprofen tablet |  |  |  |
| 1. Insulin regular injection |  |  |  |
| 1. Metformin tablet |  |  |  |
| Omeprazole tablet or alternative such as pantoprazole, rabeprazole |  |  |  |
| 1. Oral Rehydration Solution (ORS) |  |  |  |
| 1. Paracetamol tab/injection |  |  |  |
| 1. Salbutamol tab or inhaler |  |  |  |
| 1. Simvastatin tablet or other statin e.g. atorvastatin, pravastatin, fluvastatin |  |  |  |
| 1. Zinc sulphate tab |  |  |  |
